# Validation of the Animated Activity Questionnaire (AAQ) for patients with hip and knee osteoarthritis: comparison to home-recorded videos

**DOI:** 10.1007/s00296-015-3230-4

**Published:** 2015-02-14

**Authors:** W. F. Peter, M. Loos, J. van den Hoek, C. B. Terwee

**Affiliations:** 1 Department of Epidemiology and Biostatistics and the EMGO Institute for Health and Care Research, VU University Medical Centre, De Boelenlaan 1089a, 1081 HV Amsterdam, The Netherlands; 2 Amsterdam Rehabilitation Research Centre | Reade, Amsterdam, The Netherlands

**Keywords:** Questionnaire, Animations, Osteoarthritis, Validity, Activity limitations

## Abstract

A recently developed computerized Animated Activity Questionnaire (AAQ) uses video animations to measure limitations in basic daily activities in patients with hip or knee osteoarthritis (OA). The AAQ is less time and money consuming than performance-based tests and less burdensome for patients. Compared to patient-reported outcome measurements, the AAQ is less influenced by the perception of the patient. Previous validation studies of the AAQ showed promising results. Since the AAQ purports to measure activity limitations in real-life situations, this study aims to establish the construct validity of the AAQ by comparing the AAQ to home-recorded videos. Home-recorded videos of eleven basic activities performed by 22 OA patients were transformed into scores and subsequently correlated with the AAQ, the Hip disability and Knee injury Osteoarthritis Outcome Score (H/KOOS), and three performance-based tests (Stair Climbing Test, Timed Up and Go test, and the 30 s Chair Stand Test). The AAQ was expected to correlate highly with the home-recorded videos and the performance-based tests, and moderately with the H/KOOS. As hypothesized, the AAQ correlated highly with the home-recorded videos and performance-based tests (0.83 and, 0.73 respectively), but also unexpectedly highly with the H/KOOS (0.79). In conclusion, since the AAQ correlates highly with home-recorded videos, it seems to have potential as a measurement tool to assess limitations in activities close to the real-life situation, but this should be confirmed in a larger sample of patients.

## Introduction

Osteoarthritis (OA) is the most prevalent chronic joint disease worldwide, and one of the leading causes of disability and activity limitations in daily life [1, 2]. Hip and knee are two of the most affected joints [2]. An essential key in the management of hip and knee OA is a comprehensive assessment of physical limitations in daily activities [3]. Ideally, we would like to measure the level of performance of activities in daily life such as a person’s home situation. But for several logistic and privacy-related reasons, this is not possible. In general, there are two types of alternative measurements: patient-reported outcome measures (PROMs) and performance-based tests, which are both recommended to assess the patients’ level of activity limitations [4, 5]. PROMs rely on self-report and measure the experienced degree of difficulty in performing daily activities. They are considered easy to implement, inexpensive, and harmless for the patient. Nevertheless, PROMs are highly subjective to the perception of the term difficulty and to the adopted reference frame of the patient. PROM scores are influenced by a large number of personal factors (e.g., BMI, depression, self-efficacy, and pain) [6–8]. Furthermore, Fayers et al. [9] signify that some patients relate to the time before they got sick while others compare themselves to healthy peers. Furthermore, the activities referred to in the questionnaire are subject to the reference frame of the respondent [10]. For example, the perception of stair climbing highly depends on what kind of stairs a subject has in mind.

Performance-based tests, on the other hand, are associated with different limitations. They are considered expensive, time–consuming, and invasive as they demand trained personnel, test facilities, and physical presence of the patient [11]. It is also advocated that tests administered in the clinic, essentially, do not represent a real-life situation and only capture a snapshot of reality [12]. In addition to this, performance-based scores are more sensitive to observer bias, instruction bias, and the motivation of the participant to perform the test.

Research in the field encourages a complementary use of both outcome measures [6, 13, 14]. In fact, it is suggested that PROMs and performance-based test measure different constructs. Performance-based tests evaluate the maximum capacity of a patient in performing isolated tasks, while PROMs indicate the experienced degree of difficulty (i.e., a perception) [15].

No measurement tool exists that measures the actual level of performance of an activity. In light of this, the computer-administered Animated Activity Questionnaire (AAQ) was developed [16, 17]. The AAQ contains seventeen items with every item representing one basic daily activity. With the AAQ, patients watch animated videos on a computer, in which a dummy (an animation of a person) performs different activities, such as stair climbing and rising from a chair, in several ways (each way exposing a different level of difficulty). An example can be seen on: http://kmin-vumc.nl/_14_0.html. Patients are asked to select the animation that best represents their way of performing the activity.

We expect that by showing videos of performance levels instead of asking about the degree of experienced difficulty, the subjective interpretation of questions is minimized and the AAQ measures more closely the patient’s actual performance of activities in daily life.

Results of two validation studies which tested preliminary versions of the AAQ against PROMs and performance-based tests were promising [16, 17]. Currently, the cross-cultural validity of the AAQ is studied in a large international project including over 1,000 patients in seven European countries. However, since the AAQ purports to measure the performance of activities in daily life, comparisons with existing PROMs and performance-based tests will not be sufficient enough to support construct validity.

The aim of this study was to assess construct validity of the AAQ by comparing the AAQ to home-recorded videos of daily activities. Based on the construct of the AAQ, a strong correlation between the AAQ and home-recorded videos was hypothesized. Based on the fact that the AAQ measures activity limitations and not the perception of how the activities are performed, it is hypothesized that the AAQ is moderately correlated with the H/KOOS.

## Method

### Subjects

Patients were selected from two different centers: a rehabilitation centre in Amsterdam (Reade) and a physiotherapy practice in Arnhem (Formupgrade). Hip or knee OA patients were approached if they were previously (up to 12 months ago) or currently under treatment or had participated in earlier research projects (up to 12 months ago) at Reade. They were approached by phone and provided with some general information regarding the project. If a patient was interested, an information leaflet and an informed consent form were sent.

To be eligible, patients had to meet the following criteria: a diagnosis of hip or knee OA according to the clinical ACR criteria [18, 19], established by a medical doctor, general practitioner, or physiotherapist; a fluent understanding of the Dutch language and access to stairs at home or in the proximate area. In order to create a heterogeneous sample of OA patients, also patients who underwent joint replacement surgery were included in the study.

### Procedure

After informed consent was obtained, home appointments were scheduled for every individual participant. First, patients completed the AAQ and the H/KOOS ADL subscale. Both were administered at the computer in a consecutive order. Since the questionnaires are self-explanatory, no additional instructions were provided. Subsequently, videos were taken of eleven basic daily activities: rising from a chair; sitting down on a chair; walking six meters after 15 min sitting down; ascending stairs; descending stairs; sitting down on a couch; rising from a couch; rising from a toilet; sitting down on a toilet; picking something (i.e., a small coffee mug) up from the floor, and rising from the floor. Patients were instructed to perform these activities in their own home in a consecutive order and to behave as natural as possible. For this purpose, 11 of the 17 AAQ activities were selected. For practical reasons, taking of shoes and putting on shoes were not selected. After all videos were recorded, the respondents were asked to execute three performance-based tests.

### Ethical approval

This study was approved by the Medical Ethics Committee of the VU University Medical Center, Amsterdam, and conducted in accordance with the Handbook for Good Clinical Research Practice of the World Health Organization and Declaration of Helsinki principles [http://www.wma.net/en/30publications/10policies/b3/]. All patients provided written informed consent.

### Measurements

**Fig. 1:**
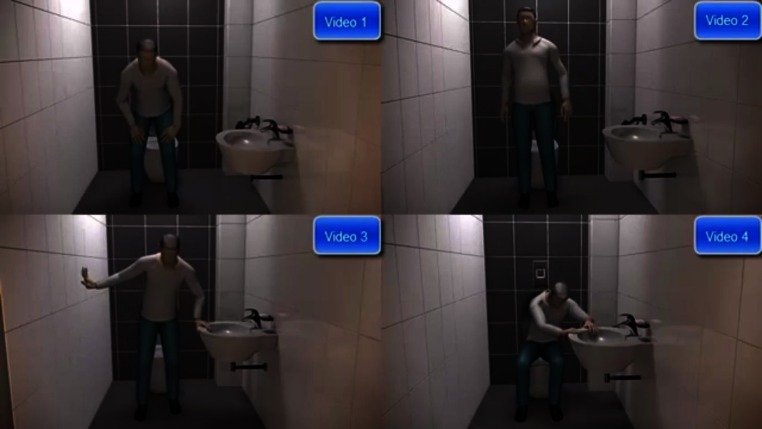
Example of a man sitting down on a toilet

**Fig. 2:**
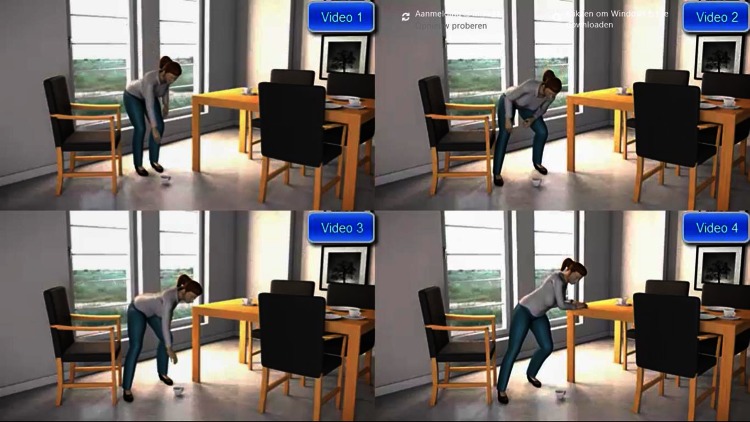
Example of a woman picking up an object from the floor

The AAQ contains seventeen activities with between three and five videos per activity. The first video of each activity represents the most optimal performance. With increasing video number, the level of difficulty in performance rises. Figures 1 and 2 show two examples, one for males and one for females. Patients are asked to choose the video that best matches their own performance of the activity. All items also offer the response option ‘unable to perform.’ Scores of the AAQ were transformed into scores on a 0–100 scale with high scores corresponding to high levels of functioning. On average, the AAQ takes 10–15 min to complete.

The home-recorded videos were independently analyzed by two physiotherapists experienced in the treatment of hip and knee OA patients. Both were not involved in the data collection and were blinded to the results of the AAQ scores, H/KOOS ADL subscale scores, and performance-based tests scores. A five-point rating scale was used to score the home-recorded videos, and the score was subsequently transformed to a 0–100 scale, with a higher score corresponding to a higher level of functioning. The five-point scale was based on the International Classification of Functioning, disability, and health (ICF) for physical impairment [15]. However, some small adjustments were made for this specific purpose. The following rating system was used: a score of (1) point indicated no problems; (2) points indicated mild problems; (3) points indicated moderate problems; (4) points indicated severe problems; and (5) points were scored if a subject was not able to perform the activity.

Details with regard to the development of the AAQ are published elsewhere [16]. A description of the activities and levels of difficulty is provided in the appendix (‘Appendix’).

Directly after completing the AAQ, patients were asked to complete a PROM; the Function, Daily Living (ADL) subscale of the HOOS [20] (i.e., Hip disability Osteoarthritis Outcome Score) or KOOS [21] (i.e., Knee injury Osteoarthritis Outcome Score). The H/KOOS ADL subscale contains seventeen physical activity-related items. Each item was rated on a five-point rating scale (i.e., 0–4). Scores were transformed in 0–100 score with higher scores corresponding to higher levels of functioning. Patients were instructed to reflect on their average disability during the last week. The applied computer program did not allow for any missing values to occur. Subsequently, patients were requested to rate their hip- or knee-related pain (i.e., on average during the last week) on a numeric rating scale, with 0 corresponding to no pain and 10 to the worst imaginable pain.

Finally, patients were asked to execute three performance-based tests in the following predetermined order: the stair climbing test (SCT) [11, 22–24], the timed up and gotest (TUG) [22, 23, 25, 26], and the 30-s repeated chair stand test (CST) [22, 23, 27, 28]. The SCT entails the timing of ascending and descending a flight of nine stairs. The TUG comprises the timing of standing up from a chair, walk three meters, turn around, walk back, and sit down again. During the CST, subjects were requested to rise and sit down on a chair as often as possible (without the support of their hands), and the number of stands within 30 s was scored.

### Statistical analysis

Descriptive statistics with regard to gender, age, joint affected (i.e., knee, hip, or both), total joint surgery (none, unilateral, bilateral), and physiotherapy treatment were presented in order to describe the study population.

Correlations were calculated between the AAQ and the home-recorded videos, the H/KOOS ADL subscale, and performance-based tests. In order to test the validity of the AAQ, the following hypothesis was formulated a priori:

1. The total score of the AAQ correlates highly (>0.60) with the total score of the home-recorded videos.

In order to compare correlations between the AAQ and home-recorded videos with the correlation between the AAQ and existing measurement instruments, the following additional hypotheses for validity were formulated:

2. The total score of the AAQ correlates highly (>0.60) with the total score of the three performance-based tests.
3. The total score of the AAQ correlates moderately (0.3–0.6) with the total score of the H/KOOS ADL subscale.
4. The correlation between the total score of the AAQ and the total score of the home-recorded videos is at least 0.10 point higher as compared to the correlation between the total score of the AAQ and the total score of the performance-based tests.
5. The correlation between the total score of the AAQ and the total score of the home-recorded videos is at least 0.20 point higher as compared to the correlation between the total score of the AAQ and total score of the H/KOOS ADL subscale.

Correlations between the AAQ and the home-recorded videos, H/KOOS ADL subscale, and performance-based tests were calculated by means of the Spearman correlation coefficients. For hypotheses 2 and 4, the correlation was calculated between the AAQ and the average score of the three performance-based tests. In order to adequately average these correlations, scores of the three different performance-based tests (i.e., SCT, TUG, and CST) were transformed into Fisher’s Z scores. After summarizing and averaging the three scores, the score was transferred back into a correlation coefficient.

## Results

Twenty-two patients participated in this study. Characteristics of the study population are presented in Table 1.

**Table 1:** Patient characteristics (*N* = 22)

Females (%)	16 (72 %)
Age [mean (SD), range]	65.3 (8.1), 50–80
BMI [mean (SD), range]	31.5 (7.2), 19.6–48.8
Years of complaints (median, range)	8.0, 3–54
Joint affected	
Knee	18
Hip	10
Hip and knee	6
Total joint replacement	
None	15
Unilateral	6
Bilateral	1
Currently under physical therapy treatment	5
AAQ score [mean (SD), range]	73.1 (17.7), 41.2–95.6
Home-recorded video score [mean (SD), range]	67. 5 (23.4), 27.3–97.7
H/KOOS ADL subscale [mean (SD), range]	56.1 (17.1), 30.6–90.3
NRS pain score [mean (SD), range]	5.9 (2.3), 1–10
Performance-based tests	
SCT (in sec., median, range)	15.5, 8.5–49.8
TUG (in sec., median, range)	10.4, 7.3–23.2
CST (median, range)	8.0, 3–17

For one subject, the video quality of the activity sitting down on a chair was not sufficient. The score for this activity was considered a missing value. The researchers assume that this missing occurred completely at random. The missing value was replaced by the mean value of the scores of the other twenty-one participants.

In Table 2, the correlations between the AAQ scores, the home-recorded video scores, the H/KOOS ADL subscale scores, and the scores of the performance-based tests are presented.

**Table 2:** Spearman correlations (95 % CI) between the total scores of the Animated Activity Questionnaire (AAQ), H/KOOS ADL subscale, and performance-based tests, in 22 patients with hips and knee osteoarthritis

	AAQ	Home-recorded videos	H/KOOS ADL subscale	Total score performance-based tests^a^	stair climbing test (SCT)	timed up and go test (TUG)	30 s chair stand test (CST)
AAQ	1.00	0.83 (0.62–0.93)	0.79 (0.56–0.91)	0.73 (0.45–0.88)	0.78 (0.54–0.91)	0.71 (0.41–0.87)	0.69 (0.38–0.869)
Home-recorded videos		1.00	0.75 (0.48–0.89)	0.83 (0.63–0.93)	0.94 (0.85–0.97)	0.77 (0.51–0.90)	0.77 (0.52–0.90)
H/KOOS ADL subscale			1.00	0.70 (0.39–0.87)	0.69 (0.38–0.86)	0.68 (0.37–0.86)	0.72 (0.43–0.88)
stair climbing test (SCT)					1.00	0.77 (0.51–0.90)	0.77 (0.51–0.90)
timed up and go test (TUG)						1.00	0.77 (0.52–0.90)
30 s chair stand test (CST)							1.00

^a^Scores based on transformation of separate performance-based tests scores into Fisher’s Z scores, calculating the average and back transformation into an average correlation score

The results in relation to our hypotheses were as follows: (1) The AAQ showed a high correlation of 0.83 with the home-recorded videos, which confirmed the hypothesis. (2) The total score of the AAQ correlated highly (0.73) with the average score of the performance-based tests, as expected (>0.60). (3) The correlation between the total score of the AAQ and the total score of the H/KOOS ADL subscale was higher (0.79) than the expected moderate correlation (0.3–0.6). (4) The difference in correlation between the AAQ and the home-recorded videos (0.83), on the one hand, and the AAQ and the performance-based tests (0.73), on the other hand, was 0.10, just as expected (difference at least 0.10 point). (5) The difference in correlation between the AAQ and the home-recorded videos (0.83), on the one hand, and AAQ and the H/KOOS ADL subscale (0.79), on the other hand, was 0.03, not as large as expected (difference at least 0.20 point).

## Discussion

The AAQ is a computerized animated measurement tool that uses videos to assess activity limitations in patients with hip and knee OA. An earlier study focused on a comparison of the AAQ with performance-based tests and self-reported questionnaires [17]. This study aimed to investigate the construct validity of the AAQ by comparing it with the patients’ real-life situation (i.e., home-recorded videos). As hypothesized, the AAQ correlated highly (0.83) with the home-recorded videos, which supports the idea that the AAQ provides an adequate representation of the performance of basic daily activities in real life. The correlation of the AAQ with the performance-based tests was also high (0.73), but the high correlation (0.79) of the AAQ with the H/KOOS ADL subscale was unexpected.

Yet, some methodological limitations might provide an explanation for the high correlations of the AAQ with the H/KOOS ADL subscale.

All tests were administered at home within a short time frame, which might elucidate small differences in correlations between the AAQ scores and scores on the home-recorded videos, the performance-based tests, and H/KOOS, respectively. The high correlation between the AAQ and the H/KOOS was also found in our earlier study [16]. In both studies, patients completed the H/KOOS directly after completing the AAQ. Patients might have been influenced by the AAQ while completing the H/KOOS ADL subscale, through the visual imprinting effect of the animations. This limitation can be tackled by changing the order of instrument administration, preferably by randomization, in future validation studies. Furthermore, the activities and number of difficulty levels of the AAQ show a strong resemblance with those of the H/KOOS ADL subscale. Another limitation of this study is the small group of 22 patients. Due to logistics and small financial resources, the minimum of 30 patients according to the COSMIN standards [29] could not be met. Therefore, conclusions must be interpreted with caution and the study must be repeated in a larger sample size. Finally, no inter-rater reliability was tested in the observers while judging scores on the home-recorded videos.

The application of video animations for the measurement of activity limitations remains an unexplored area. Recently, Rejeski and colleagues developed a measurement instrument that uses video animations to describe a large set of physical challenges [30]. They also reported high correlations (0.58 and 0.59) between the video animation and performance-based tests, which supports the idea that video animations can be a good alternative for performance-based tests. Compared to the AAQ, however, this instrument contains an essential difference. For every activity, only one video is shown. Subsequently, the patient is asked whether or how many times he or she is able to repeat the displayed performance. Rejeski et al. also provided evidence that varying contextual features (i.e., stair climbing with or without a handrail) influences persons’ perception of their abilities [10]. We expect that by showing similar contextual features in different levels of performance of each activity, the AAQ will be less influenced by perception.

Besides the unique construct of the AAQ, there are other features that support a clinical implementation. In comparison with performance-based tests, the AAQ is less exposed to observer bias, bias by different test instructors, and the motivation of the patient to perform the test. And since all items refer to the average performance during the last week, the AAQ does not cover only one moment in time, as performance-based tests. Furthermore, the AAQ is considered relatively cheap, easy to implement, and less burdensome for the patient.

When compared to PROMs, the AAQ also has additional benefits. The AAQ refers to videos of the performed activities instead of the word difficulty which reduces the impact of different reference frames along with ambiguities regarding the interpretation of the term difficulty. Moreover, all videos offer a standardized virtual reality environment, which reduces the impact of different environments on the subjects’ rating. Additionally, only a limited amount of text is included, which facilitates an international implementation of the AAQ, since minimal translation is needed. Finally, minimal reading ability is required.

Nevertheless, as the AAQ relies on self-report, it will be influenced by some personal factors such as self-confidence, self-efficacy, and the ability to self-reflect. The standardized virtual environment displayed in the video is not always specific to the subjects’ home situation. And it is debatable if all patients are able to self-reflect in completing a questionnaire based on video animations.

Before the AAQ can be implemented in clinical practice, more research is necessary. First, the structural validity has to be established by use of factor analysis or item response theory analyses. Next, a larger study with a more optimal design should be carried out in order to investigate the role of potential contamination between results of the AAQ and H/KOOS ADL subscale. For instance, the order in which the AAQ and H/KOOS was administered could be reversed or randomized in a future study. Furthermore, the performance-based tests need to be administered in the clinic and not in a subjects’ home environment. Since the AAQ has a potential value for international use because minimal translation is needed, further research should focus on cross-cultural validity.

In conclusion, since the AAQ correlates highly with home-recorded videos, it seems to have potential as a measurement tool to assess limitations in activities close to the real-life situation, although this should be confirmed in a larger study.

## Appendix

See Table 3.

**Table 3:** Set of activities of the AAQ with levels of execution

Basic set of activities (17 activities and 68 videos)	
1	Ascending stairs
	Setting: stairs with banister
	Without any problem, touching the banister but not leaning
	With slower speed, and pulling up at the banister
	One stair step at the time and pulling up at the banister
	One stair step at the time and pulling up at the banister and using one crutch and rest in between
	Unable to perform
2	Descending stairs
	Setting: stairs with banister
	Without any problem, touching the banister but not leaning
	With slower speed, a little bit leaning on the banister
	One stair step at the time and with more support of the banister
	One stair step at the time and leaning on the banister and using one crutch
	One stair step at the time but going backwards and holding on to the banister
	Unable to perform
3	Walking outside on a flat surface
	Setting: outside on the sidewalk
	Without any problems
	With slower speed and shorter stand phase of affected leg
	Shorter stand phase and longer sway phase injured leg and a Duchenne gait
	With help from a stick or crutch
	Shorter stand phase and longer sway phase injured leg and a walker
	Unable to perform
4	Walking outside on oneven terrain
	Settings: a forest
	Without any problems
	With slower speed and shorter stand phase of affected leg
	Shorter stand phase and longer sway phase injured leg and a Duchenne gait
	With help from a stick or crutch
	Shorter stand phase and longer sway phase injured leg and a walker
	Unable to perform
5	Walking inside: starting walking after at least 15 min sitting
	Setting: living room with ordinary chair
	Without any problem
	First making some steps on the place and then starting slowly with shorter stand phase later normal stand phase
	With slower speed and starting with smaller steps
	With slower speed, starting in more bending position and slowly stretching up the body during the first meters and walking with a Duchenne gait
	Unable to perform
6	Ascending a bridge
	Setting: small bridge and water
	Without any problems
	With slower speed and shorter stand phase of affected leg
	With help from a stick or crutch
	Shorter stand phase and longer sway phase injured leg and a walker
	Unable to perform
7	Descending a bridge
	Setting: small bridge and water
	Without any problems
	With slower speed and shorter stand phase of affected leg
	With help from a stick or crutch
	Shorter stand phase and longer sway phase injured leg and a walker
	Unable to perform
8	Picking up an object from the floor
	Without any problem with bending knees
	With slower speed supported by leaning with the forearm on the thigh, legs somewhat spread
	Bending with lower back and little flexion in knees and spread legs and more support on good leg. Index leg aside
	Leaning on chair or other object, spread legs with more weight on good leg and index leg aside
	Unable to perform
9	Rising from the floor
	Setting: room with table/chair
	Turning to the belly side and get into hands/knees position. Subsequently lifting the good leg and leaning on the foot and lifting up oneself by pushing with hands on the knee
	Turning to the belly side and get into hands/knees position, spreading the legs. Subsequently coming to hands/feet position and ‘walking’ with hands toward feet, leaning first with one hand and then with two hands on the legs and pushing oneself to a standing position
	Turning to the belly side and get into hands/knees position. Subsequently standing up with the help of a chair or small cabinet to standing position, while leaning with the hands or forearms
	Unable to perform
10	Rising from a chair
	Setting: room with table/chair
	Without any problem
	With lower speed, leaning with hands on arm rest and with more weight on the good leg
	Leaning on both hands, bending forward and pushing oneself upwards, stretching legs first, and secondly stretching up the upper body
	Scrolling forward on the chair, moving forward/backward with the upper body to make some speed to get up. When getting up pushing oneself with the hands on the arm rests, first stretching the legs with the upper body in bending position and subsequently erect the whole body
	Unable to perform
11	Sitting down on a chair
	Setting: room with table/chair
	Without any problem
	With slower speed, leaning with the hands on the arm rests and more weight on the good leg
	Leaning on both hands, bending forward and letting down oneself, leaning on the hands and with the affected leg more in forward and stretched position
	The same as above but with plopping down in the chair during the last phase of the movement
	Unable to perform
12	Rising from a sofa
	Setting: room with table/sofa
	Without any problem
	With lower speed, leaning with one hand on arm rest and with the other on the sofa and with more weight on the good leg
	The same as above but now also bending forward and pushing oneself upwards, stretching legs first and secondly stretching up the upper body
	Scrolling forward and moving forward/backward with the upper body to make some speed to get up. When getting up pushing oneself with the hands on the sofa, first stretching the legs with the upper body in bending position and subsequently erect the whole body
	Unable to perform
13	Sitting down on a sofa
	Setting: room with table/chair
	Without any problem
	With slower speed, leaning with one hand on the arm rest and the other on the sofa and more weight on the good leg
	Leaning on both hands, bending forward and letting down oneself, leaning on the hands and with the affected leg more in forward and stretched position
	The same as above but with plopping down on the sofa during the last phase of the movement
	Unable to perform
14	Rising from a toilet
	Setting: toilet with a small fountain and holder for toilet paper
	Without any problem
	With support of leaning on the toilet on which they are sitting and good bending forward with the upper body
	Rise with support of pulling oneself up at the small fountain, slower speed and taking some time to find balance
	Moving forward/backward with the upper body to make some speed to get up. Rise with support of pulling oneself up at the small fountain
	Unable to perform
15	Sitting down on a toilet
	Setting: toilet with a small fountain and holder for toilet paper
	Without any problem
	With support of leaning on the toilet and bending forward while getting to be seated
	The same but with some hanging on the small fountain
	Slowly while hanging a bit on the small fountain and plopping down during the last phase of the movement
	Unable to perform
16	Putting on shoes
	Setting: bedroom with bed and night cabinet
	Doing it while sitting and pulling up one leg
	Doing it while sitting and leaning with one leg (ankle) on the other leg (knee)
	Doing it sitting bending forward and leave the feet on the ground
	With help from a assistive device
	Unable to perform
17	Taking of shoes
	Setting: bedroom with bed and night cabinet
	Doing it standing taking of shoe with the help of the other foot
	While sitting, pulling up the leg/foot, loosening laces, and then taking of shoe with the help of the other foot
	While sitting, bending forward, keeping the foot on the floor, loosening laces, and taking of the shoe with the other foot
	Unable to perform
